# A new species of *Melita* from Japan (Crustacea, Amphipoda, Melitidae)

**DOI:** 10.3897/zookeys.760.24778

**Published:** 2018-05-28

**Authors:** Ko Tomikawa, Kentaro Hirashima, Atsushi Hirai, Ryu Uchiyama

**Affiliations:** 1 Department of Science Education, Graduate School of Education, Hiroshima University, 1-1-1 Kagamiyama, Higashihiroshima, Hiroshima 739-8524, Japan; 2 Wakayama Prefectural Museum of Natural History, 370–1, Funo, Kainan, Wakayama 642–0001, Japan; 3 Susami Town Aquarium of Prawns and Crabs, 808-1 Sumie, Susami, Wakayama 649-3142, Japan

**Keywords:** Brackish water, Choshi River, COI, Mie Prefecture, taxonomy

## Abstract

A new brackish-water species of melitid amphipod, *Melita choshigawaensis*, from the Choshigawa River, Mie Prefecture, Japan, is named and described. *Melita choshigawaensis* **sp. n.** is distinguished from the most similar *M. shimizui* (Uéno, 1940) by having an elongate and weakly arched male uropod 3, and a deep and strongly hooked anterior lobe of the coxa on the female’s pereopod 6. Nucleotide sequences of the mitochondrial cytochrome *c* oxidase subunit I (COI) of *M. choshigawaensis* and *M. shimizui* support divergence at the species level. A key to the Japanese species of *Melita* is provided.

## Introduction

The amphipod genus *Melita* Leach, 1814 comprises approximately 80 species worldwide (Krapp-Schickel and Sket 2015), most of which occur in marine intertidal and shallow waters, though some inhabit brackish and freshwaters (Jarrett and Bousfield 1996; Krapp-Schickel and Sket 2015). Eleven species of *Melita* have been recorded from Japan: *M. bingoensis* Yamato, 1987; *M. hoshinoi* Yamato, 1990; *M. koreana* Stephensen, 1944; *M. longidactyla* Hirayama, 1987; *M. nagatai* Yamato, 1987; *M. pilopropoda* Hirayama, 1987; *M. quadridentata* Yamato, 1990; *M. rylovae* Bulycheva, 1955; *M. setiflagella* Yamato, 1988; *M. shimizui* (Uéno, 1940); *M. tuberculata* Nagata, 1965. Among them, two species, *M. setiflagella* and *M. shimizui*, are known from brackish lakes and river mouths (Nagata 1965; Hirayama 1987; Yamato 1987, 1988, 1990; Ishimaru 1994). However, it is apparent that the diversity of species of *Melita* in Japanese waters, particularly in brackish environments, is not fully appreciated.

During field surveys of aquatic fish and amphipod faunas in the Choshi River, Mie Prefecture, Japan, a new amphipod species was found. Though DNA nucleotide sequence data have been recently successfully used to differentiate morphologically similar amphipod species (Matsukami et al. 2017; Tomikawa et al. 2016, 2017), previous taxonomic studies on *Melita* in Japan have focused on morphological characteristics only. Here, both molecular and morphological data are used to differentiate this species from others, which is described and illustrated. A key to species of *Melita* in Japanese waters using conventional morphological criteria is provided.

## Materials and methods

### Specimens

**Figure 1.**
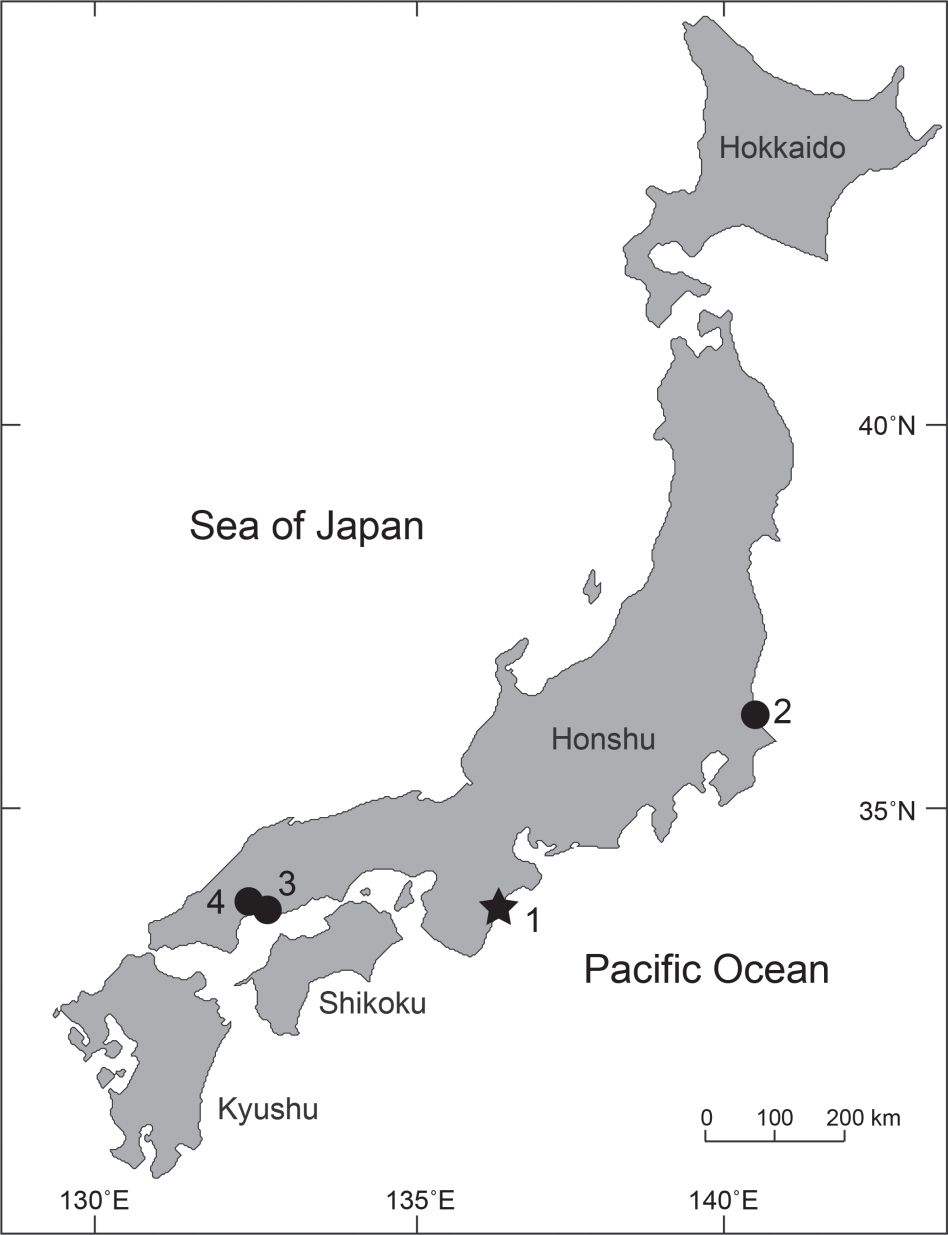
Collection locations: *M. choshigawaensis* sp. n. (★) and *M. shimizui* (●). Names of localities are shown in Table 1.

Specimens were collected using a hand net (mouth 25 cm wide, 17 cm high, mesh size 0.1–0.5 mm) from under stones at the mouth of Choshi River, Kihoku, Mie Prefecture (Fig. 1), before being fixed in 99% ethanol. The specimens have been deposited in the National Museum of Nature and Science, Tsukuba (NSMT)

**Table 1.** Uncorrected *p*-distances (%) of COI sequences (658 bp) among *M. choshigawaensis* sp. n. and three populations of *M. shimizui* (Uéno, 1940). Numbers after localities correspond to locations in Figure 1.

	Species	Locality	1	2	3	4
1	*M. choshigawaensis* sp. n.	Choshi River, Mie (1)	0.0–0.2			
2	*M. shimizui* (Uéno, 1940)	Lake Hinuma, Ibaraki (2)	14.9	–		
3		Seno River, Hiroshima (3)	14.9	4.4	–	
4		Ota River, Hiroshima (4)	14.9	4.4	0.0	–

### Morphological observation

All appendages were dissected in 80% ethanol and mounted in gum-chloral medium on glass slides using a stereomicroscope (Olympus SZX7). Slides were examined using a light microscope (Nikon Eclipse Ni), with appendages illustrated using a camera lucida. Body length (BL, to the nearest 0.1 mm) was measured from the rostrum tip to the telson base, along the dorsal curvature. Type specimens are deposited at the National Museum of Nature and Science, Tsukuba (NSMT).

### PCR and DNA sequencing

Genomic DNA extraction from body or appendage muscle followed Tomikawa et al. (2014). The cytochrome *c* oxidase subunit I (COI) gene [LCO1490 and HCO2198 (Folmer et al. 1994)] primer set was used for PCR and cycle sequencing (CS) reactions. PCR reactions and DNA sequencing were performed following Tomikawa et al. (2017). PCR reactions were performed using a PC-320 thermal cycler (ASTEC) with an Ex *Taq* Polymerase Kit (Takara Bio Inc.). PCR mixtures were heated to 94 °C for 7 min, followed by 35 cycles at 94 °C (45 s), 42 °C (1 min), and 72 °C (1 min), and a final extension at 72 °C for 7 min. Amplification products were purified using the silica method (Boom et al. 1990). All sequencing reactions were performed according to the manufacturer’s instructions using the BigDye Terminator v3.1 Cycle Sequencing Reaction Kit (Applied Biosystems, Foster City, CA). Cycle sequencing conditions were 25 cycles of 10 s at 96 °C, 5 s at 50 °C, and 4 min at 60 °C. Sequencing reaction products were purified by ethanol precipitation. Labeled fragments were analyzed using an ABI 3130x Genetic Analyzer (Applied Biosystem). Sequences obtained from both strands of gene segments (for verification using the same primers) were edited using MEGA7 (Kumar et al. 2016). DNA sequences have been deposited with the International Nucleotide Sequence Database Collaboration (INSDC) through the DNA Data Bank of Japan (DDBJ).

## Taxonomy

### Melitidae Bousfield, 1973

#### *Melita* Leach, 1814

##### Melita choshigawaensis sp. n.

Taxon classificationAnimaliaAmphipodaMelitidae

http://zoobank.org/C10A0F95-5419-4534-8923-07D8C2E77F17

**Figure 2.**
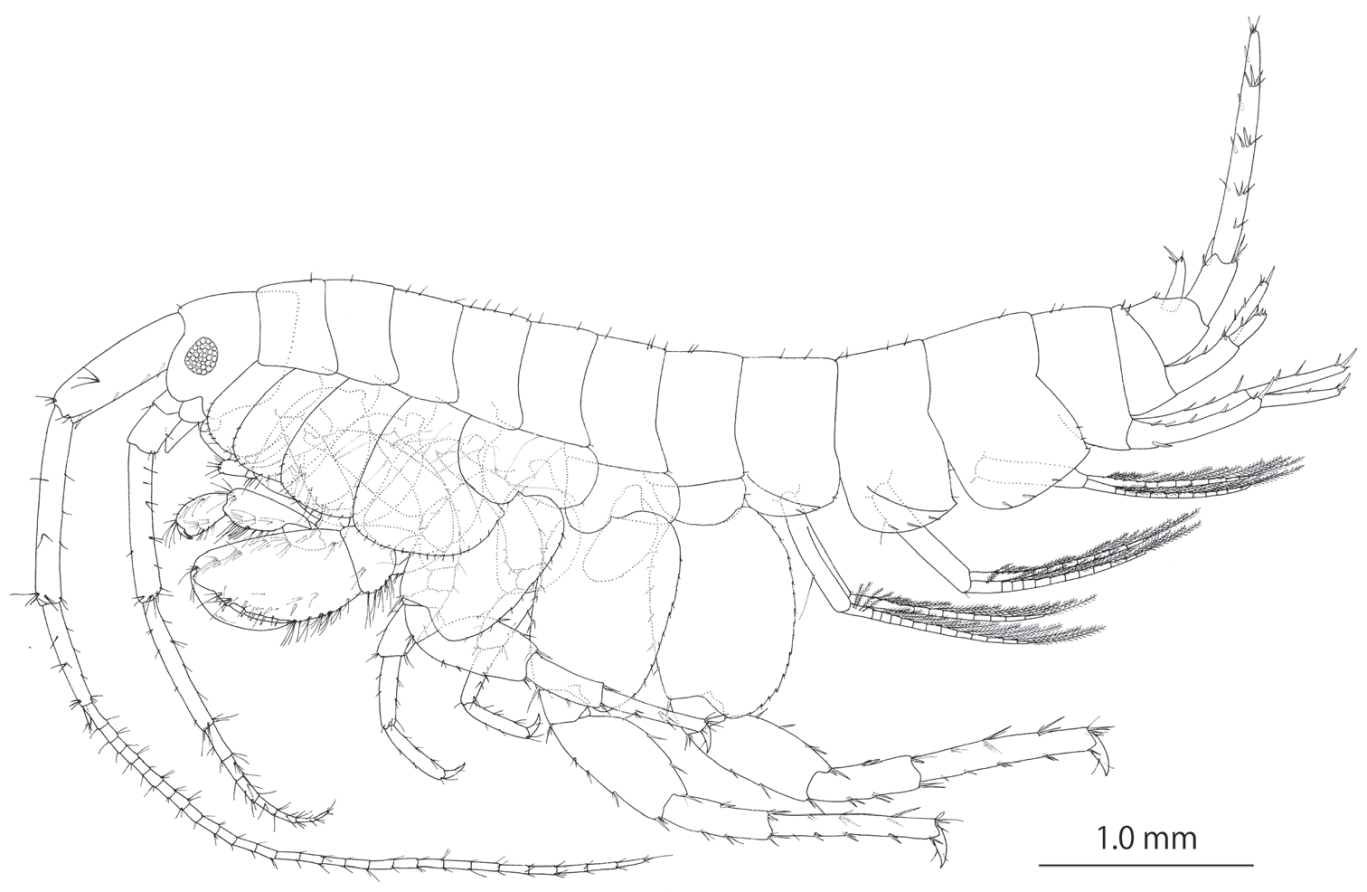
*Melita choshigawaensis* sp. n., holotype, male, BL 5.3 mm, NSMT-Cr 25826, Choshi River, Kihoku, Mie Prefecture, Japan. Habitus, lateral view.

**Figure 3.**
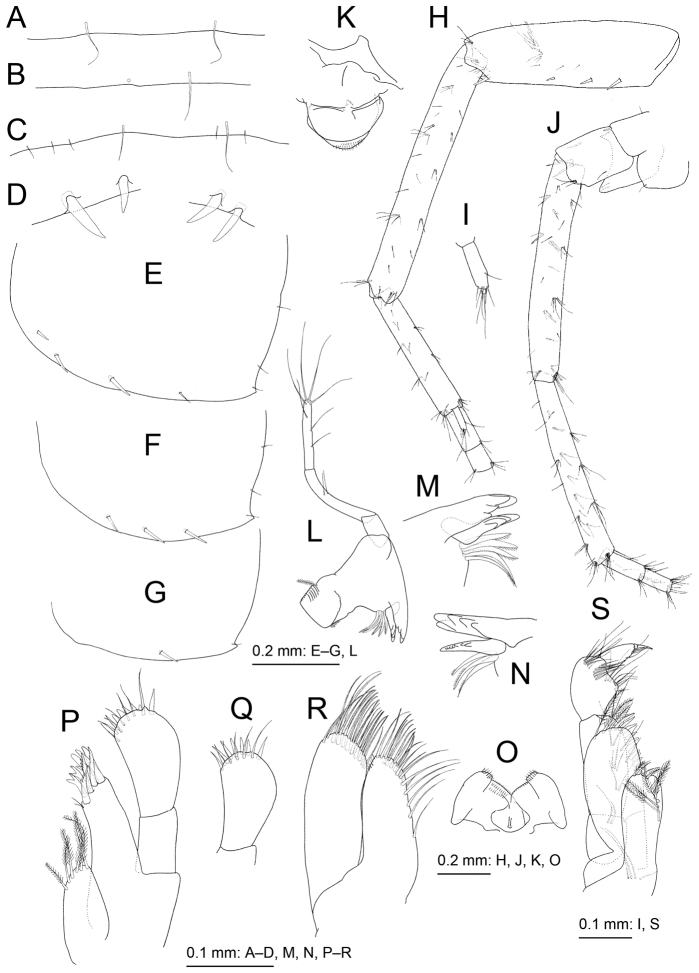
*Melita choshigawaensis* sp. n., holotype, male, BL 5.3 mm, NSMT-Cr 25826, Choshi River, Kihoku, Mie Prefecture, Japan. **A–C** dorsal margins of pleonites 1–3, dorsal views **D** dorsal margin of urosomite 2, dorsal view **E–G** epimeral plates 1–3, lateral views **H** right antenna 1, medial view, some articles of main flagellum omitted **I** accessory flagellum of right antenna 1, medial view **J** right antenna 2, medial view, some articles of flagellum omitted **K** upper lip, anterior view **L** left mandible, medial view **M** incisor, lacinia mobilis, and accessory setal row of left mandible, medial view **N** incisor, lacinia mobilis, and accessory setal row of right mandible, medial view **O** lower lip, ventral view **P** right maxilla 1, anterior view **Q** palp article 2 of left maxilla 1, posterior view **R** left maxilla 2, anterior view **S** left maxilliped, anterior view.

**Figure 4.**
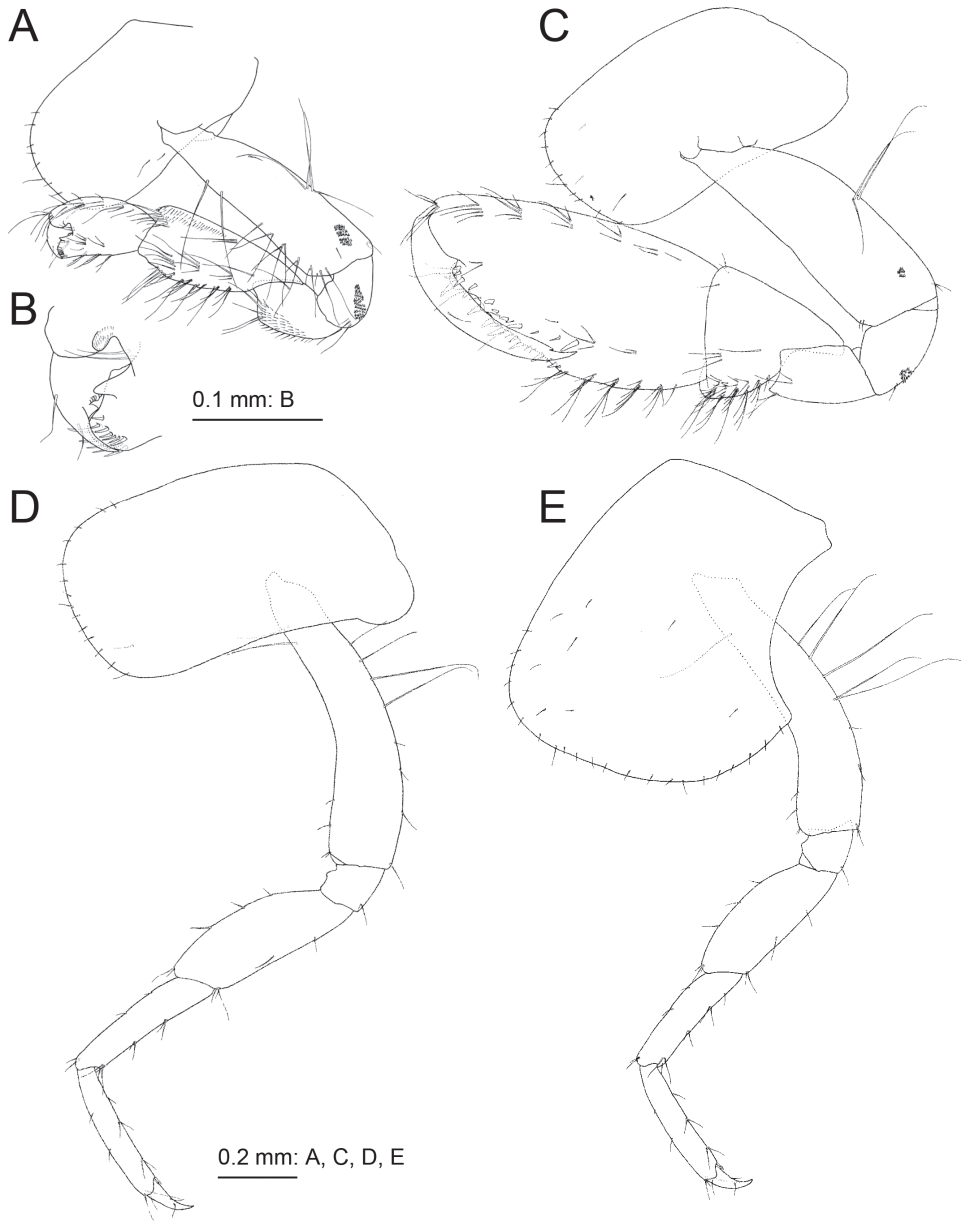
*Melita choshigawaensis* sp. n., holotype, male, BL 5.3 mm, NSMT-Cr 25826, Choshi River, Kihoku, Mie Prefecture, Japan. **A** right gnathopod 1, medial view **B** palmar margin of propodus and dactylus of right gnathopod 1, medial view **C** right gnathopod 2, medial view **D** left pereopod 3, lateral view **E** left pereopod 4, lateral view.

**Figure 5.**
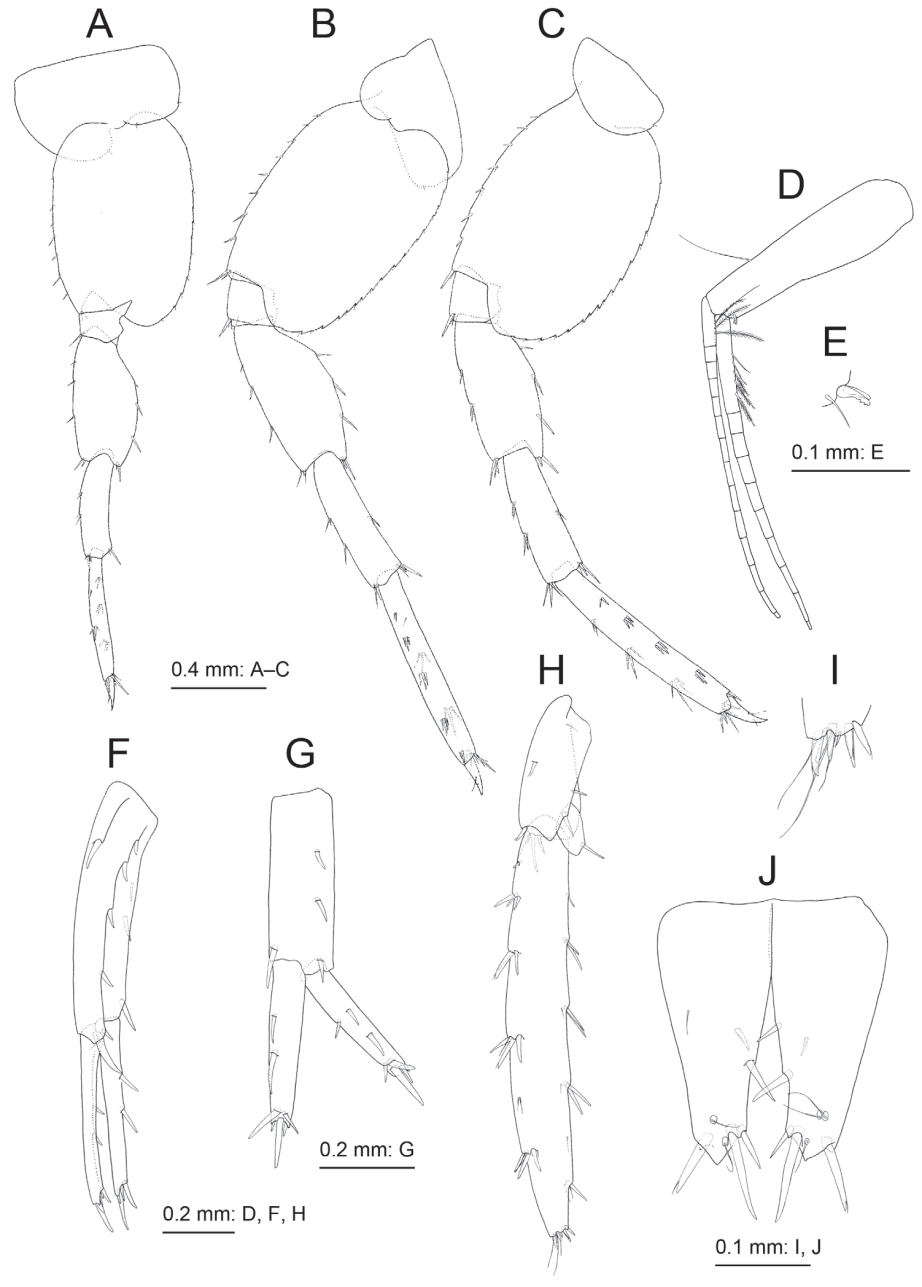
*Melita choshigawaensis* sp. n., holotype, male, BL 5.3 mm, NSMT-Cr 25826, Choshi River, Kihoku, Mie Prefecture, Japan. **A** right pereopod 5, medial view; **B** left pereopod 6, lateral view **C** left pereopod 7, lateral view **D** pleopod 1, medial view, some setae on rami omitted **E** retinacula on peduncle of pleopod 1 and associated seta, medial view **F** left uropod 1, dorsal view **G** right uropod 2, dorsal view **H** left uropod 3, dorsal view **I** distal part of outer ramus of left uropod 3, dorsal view **J** telson, dorsal view.

**Figure 6.**
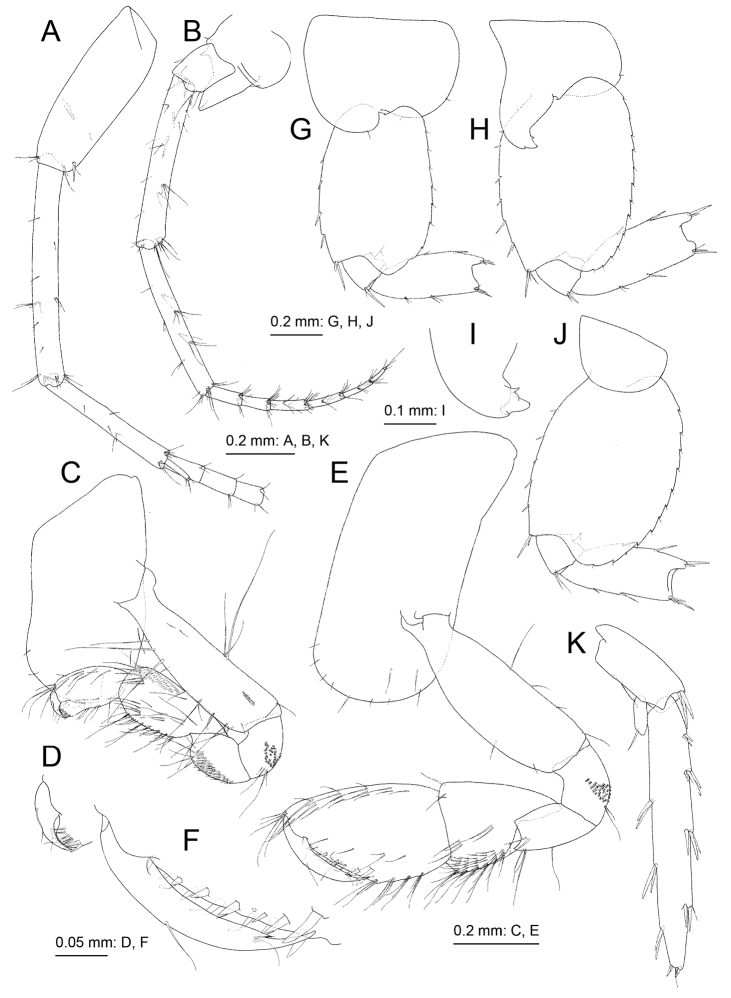
*Melita choshigawaensis* sp. n., paratype, female, BL 5.8 mm, NSMT-Cr 25829, Choshi River, Kihoku, Mie Prefecture, Japan. **A** right antenna 1, medial view, some articles of main flagellum omitted **B** right antenna 2, medial view; **C** right gnathopod 1, medial view **D** palmar margin of propodus and dactylus of right gnathopod 1, medial view **E** right gnathopod 2, medial view **F** palmar margin of propodus and dactylus of right gnathopod 2 **G** left pereopod 5, lateral view, carpus–dactylus omitted **H** left pereopod 6, lateral view, carpus–dactylus omitted **I** distal part of coxa anterior lobe of left pereopod 6, lateral view **J** left pereopod 7, lateral view, carpus–dactylus omitted; **K** right uropod 3, dorsal views.

**Figure 7.**
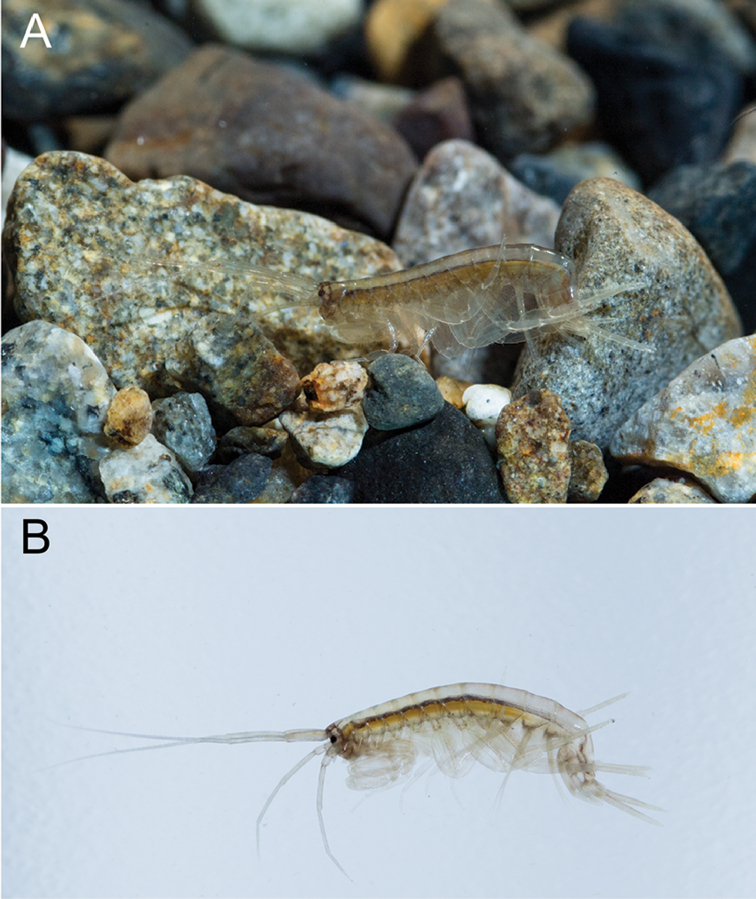
*Melita choshigawaensis* sp. n. **A, B** live males, BL *ca* 6 mm, lateral views. Photographed by Ryu Uchiyama.

Figures 2 , 3 , 4 , 5 , 6 , 7 New Japanese name: Choshigawamerita-yokoebi

###### Material examined.

Holotype: male (BL 5.3 mm, NSMT-Cr 25826), Choshi River, Kihoku, Mie Prefecture, Japan (34.108242°N, 136.221998°E), col. Ko Tomikawa, Kentaro Hirashima, Atsushi Hirai, and Ryu Uchiyama, 2 March 2017. Paratypes: male (BL 4.1 mm, NSMT-Cr. 25827), data as for holotype; male (BL 6.8 mm, NSMT-Cr. 25828), female (BL 5.8 mm, NSMT-Cr. 25829), 27 December 2017, locality and collectors as for holotype.

###### Diagnosis.

Male gnathopod 2 propodus with oblique palmar margin; anterior lobe of female pereopod 6 coxa deep and strongly hooked; male uropod 3 outer ramus uni-articulate, weakly arched, its length 7–8 times its width, lacking long setae.

###### Description male

**(holotype, NSMT-Cr 25826).** Head (Fig. 2) slightly shorter than pereonites 1 and 2 combined; rostrum short; eyes ovate; lateral cephalic lobe rounded; antennal sinus quadrate, not incised. Pereonites 1–7 (Fig. 2) dorsally smooth with fine setae. Dorsal margins of pleonites 1–3 (Fig. 3A–C) with 2, 2, and 7 setae, respectively; epimeral plate 1 (Fig. 3E) ventral submargin with three robust and one slender setae, posterior margin with two setae, posterodistal corner weakly pointed with seta; epimeral plate 2 (Fig. 3F) ventral margin with three robust setae, posterior margin with two setae, posterodistal corner pointed with seta; epimeral plate 3 (Fig. 3G) ventral margin with robust seta, posterior margin bare, posterodistal corner weakly pointed with seta. Dorsal margin of urosomite 2 (Fig. 3D) with four robust setae.

Antenna 1 (Fig. 3H): length 1.1 times that of body; length ratio of peduncular articles 1–3 as 1.0:1.3:0.7; ventral margin of peduncular article 1 with three robust setae, posterodistal corner with robust seta; primary flagellum 28-articulate with a few setae; accessory flagellum (Fig. 3I) 2-articulare, with short terminal article. Antenna 2 (Fig. 3J) half of antenna 1 length; peduncular article 5 length 0.9 times that of article 4; flagellum 7-articulate, article 1 length 1.5 times that of article 2; calceoli absent.

Upper lip (Fig. 3K) ventral margin convex, rounded, with minute setae. Left and right mandibular incisors (Fig. 3L–N) 4- and 5-dentate, respectively, with left lacinia mobilis quadri-dentate (Fig. 3M) and right (Fig. 3N) multidentate; left and right accessory setal rows (Fig. 3M, N) with five and three bladed setae, respectively; molar process triturative with plumose seta; palp tri-articulate, length ratio of articles 1–3 1.0:2.3:2.0, article 1 bare, article 2 with two setae, article 3 with seven setae. Lower lip (Fig. 3O) outer lobes broad, setulose, mandibular lobes narrow; inner lobes distinct. Maxilla 1 (Fig. 3P, Q) inner plate narrow with six plumose setae; outer plate rectangular with nine serrate robust setae; palp 2-articulate; article 1 rectangular, lacking setae; article 2 expanded, outer margin without setae, apical margin with robust and slender setae. Maxilla 2 (Fig. 3R) inner plate with oblique inner row of seven setae; outer plate slightly longer than inner plate. Maxilliped (Fig. 3S) distal part of inner plate not reaching half of palp article 2; outer plate ovate, exceeding half of palp article 2, apical margin with plumose setae, inner submargin with robust setae; palp quadri-articulate, article 4 with nail.

Gnathopod 1 (Fig. 4A, B) smaller than gnathopod 2; ventral margin and posterior submargin of coxa with setae; basis, anterior and posterior margins with long setae, posterodistal submargin with tiny palmate setae; ischium with tiny palmate setae; merus with small ventral setae; carpus not lobate, length 1.5 times that of propodus, anterior submargin with small setae, posterior margin with clusters of setae; propodus without anterodistal hood, palmar margin convex with two rows of robust setae, proximal part of palmar margin with distinct protuberance; dactylus short, not exceeding palmar margin. Gnathopod 2 (Fig. 4C) coxa subrectangular, ventral margin and submargin with setae; basis anterior margin bare, posterior margin with long setae, antero- and posterodistal corners with small setae, posterodistal submargin with small palmate setae; carpus not lobate, length 0.5 times that of propodus; propodus large, half as wide as long, palmar margin oblique with nine medial and ten lateral robust setae; dactylus with small posterodistal notch, of similar length to palmar margin.

Pereopod 3 (Fig. 4D) coxa subrectangular, ventral margin and submargin with setae; basis arched, anterior and posterior margins with long and short setae; length ratio of merus, carpus, propodus and dactylus 1.0:0.9:0.8:0.3. Pereopod 4 (Fig. 4E): coxa expanded with posterior concavity, bearing ventral and surface setae; basis anterior and posterior margins with long and short setae; length ratio of merus, carpus, propodus and dactylus 1.0:0.9:0.8:0.3. Pereopod 5 (Fig. 5A) coxa bilobate, anterior lobe large with small seta on distal margin, posterior lobe with small setae on ventral margin and posterodistal corner; basis with posterodistal lobe; length ratio of merus, carpus, propodus and dactylus 1.0:0.8:0.9:0.2; merus weakly expanded, half as wide as long. Pereopod 6 (Fig. 5B) coxa bilobate, shallower than that of pereopod 5, posterior lobe with small seta on posterodistal corner; basis posterior margin weakly serrate, posterodistal corner lobate; length ratio of merus, carpus, propodus and dactylus 1.0:0.9:1.3:0.3; merus weakly expanded, half as wide as long. Pereopod 7 (Fig. 5C) coxa semicircular, with seta on posterior margin; basis subovate, posterior margin weakly serrate, bearing posterodistal lobe; length ratio of merus, carpus, propodus and dactylus 1.0: 0.8:1.2:0.3; merus 0.4 times as wide as long.

Coxal gills (Fig. 2) present on gnathopod 2, and pereopods 3–6.

Pleopod 1–3 (Fig. 5D) peduncles with paired retinacula (Fig. 5E) on inner distal margin, and bifid plumose setae (clothes-pin setae) on inner ramus inner basal margin.

Uropod 1 (Fig. 5F) extending beyond uropod 2; peduncle with basofacial seta; inner ramus length 0.6 times that of peduncle, with two inner marginal and four distal robust setae, proximal part with slender seta; outer ramus 1.1 times longer than inner ramus, bearing two outer marginal and four distal robust setae. Uropod 2 (Fig. 5G) not extending beyond peduncle of uropod 3; inner ramus 0.9 times as long as peduncle, with two inner robust setae, distal part with five robust setae; outer ramus 0.9 times as long as inner ramus, with one inner and two outer robust setae, distal part with four robust setae. Uropod 3 (Fig. 5H, I) peduncle extending beyond telson; inner ramus length 0.13 times that of outer ramus, with distal robust seta; outer ramus with single article, weakly arched, length 2.9 times that of peduncle and 7.0 times that of outer ramus width, long setae absent. Telson (Fig. 5J) length 1.1 times longer than wide, completely cleft, each lobe with two lateral and three distal robust setae.

###### Description female

**(paratype, NSMT-Cr 25829).** Antenna 1 (Fig. 6A) 0.6 times body length; length ratio of peduncle articles 1–3 1.0:1.2:0.7; ventral margin of peduncular article 1 without robust setae; primary flagellum 17-articulate. Antenna 2 (Fig. 6B) length half that of antenna 1.

Gnathopod 1 (Fig. 6C, D) coxa elongate, anterior margin weakly concave; carpus length 1.6 times that of propodus; proximal part of palmar margin without protuberance. Gnathopod 2 (Fig. 6E, F): coxa elongate; anterior margin of basis with seta; carpus length 0.8 times that of propodus; propodus 0.6 times as wide as long, palmar margin with six medial and six lateral robust setae.

Pereopods 5–7 (Fig. 6G, H, J). Depth of pereopod 6 (Fig. 6I) anterior lobe equal to coxal width, strongly hooked; merus width 0.4 times that of length.

Uropod 3 (Fig. 6K) inner ramus length 0.15 times that of outer ramus; outer ramus sublinear, length 2.5 times that of peduncle and 6.6 times outer ramus width.

16 eggs.

###### Variation.

Uropod 3 outer ramus length 2.8 times that of peduncle and 8.2 times outer ramus width (male 6.8 mm, NSMT-Cr 25828).

###### Sequences and COI genetic distances.

In total, 658 bp of six nucleotide sequences were determined: paratypes of *M. choshigawaensis* sp. n. (NSMT-Cr 25827–25829), three sequences (LC371923–371925); and *M. shimizui* from three localities, one from Lake Hinuma (LC371926), one from Seno River (LC371927), and one from Ota River (LC371928). Uncorrected *p*-distances between *M. choshigawaensis* and *M. shimizui* were 14.9% (Table 1). Intraspecific distances of *M. choshigawaensis* and *M. shimizui* were up to 0.2% and 4.4%, respectively (Table 1).

###### Distribution.

Known only from the type locality.

###### Etymology.

Derived from the name of the type locality.

###### Remarks.

*Melita choshigawaensis* is closely related to *M. shimizui* (Uéno, 1940), originally described from a freshwater pond on Liaodong Peninsula, China (Uéno 1940), but subsequently recorded from several brackish sites in the Japanese archipelago, such as Honshu, Shikoku, Kyushu, and the main island of Okinawa (Yamato 1988). Recently, Labay (2016) described a new subspecies, *M. shimizui sakhalinensis* from Sakhalin. The pleonites of both species lack dorsal teeth, urosomite 2 has robust setae on the dorsal margin, the accessory flagellum of antenna 1 is bi-articulate, and the outer ramus of uropod 3 is uni-articulate and lacks long setae. However, *M. choshigawaensis* can be distinguished from *M. shimizui* by (features of *M. shimizui* in parentheses): the outer ramus of male uropod 3 being weakly arched (compared with sublinear) and more than seven times longer than wide (ca. 5), and the anterior lobe of the female pereopod 6 coxa is deep, equal in length to coxal width (shorter than width), and strongly (as opposed to weakly) hooked. These two species also differ genetically in COI (14.9%) greater than distances (3.5–4%) proposed as thresholds for amphipod species discrimination (Witt et al. 2006; Rock et al. 2007; Hou et al. 2009). Thus, we determined *M. choshigawaensis* represented a novel species.

*Melita choshigawaensis* is similar to *M. laevidorsum* Stephensen, 1944 from Korea, and *M. myersi* Karaman, 1987 from Australia, Fiji, and New Caledonia in that all three have dorsally smooth pleonites, a urosomite 2 with robust setae on their dorsal margins, and an elongate outer ramus of uropod 3 (Stephensen 1944; Karaman 1987). However, *M. choshigawaensis* differs from *M. laevidorsum* in having an accessory flagellum of antenna 1 with two articles (compared with four), in lacking an anterodistal hood on the propodus of male gnathopod 1 (compared with having one), and in that the medial surface of the propodus of male gnathopod 2 is sparsely (as opposed to densely) setose. From *M. myersi*, *M. choshigawaensis* differs in having a deep antennal sinus (compared with shallow), in lacking an anterodistal hood on the propodus of the male’s gnathopod 1 (compared with having one), and in having the meri of pereopods 5 and 6 weakly expanded (as opposed to their not being expanded).

#### Key to species of *Melita* in Japan

Since records of three species, *M. coroninii* Heller, 1867, *M. dentata* (Krøyer, 1842), and *M. palmata* (Montagu, 1804) from Japanese waters are dubious (Ishimaru 1994), these species are excluded from the key.

**Table:** 

1	Uropod 3, outer ramus 1-articulate	**2**
‒	Uropod 3, outer ramus 2-articulate	**9**
2	Pleonites 1–3 each with dorsal tooth	***M. tuberculata* Nagata, 1965**
‒	Pleonites 1–3 dorsally smooth	**3**
3	Dactylus of pereopods 3 and 4 long, feeble	***M. longidactyla* Hirayama, 1987**
‒	Dactylus of pereopods 3 and 4 short, stout	**4**
4	Urosomite 2 with teeth	**5**
‒	Urosomite 2 without teeth	**6**
5	Female pereopod 6, anterior lobe of coxa shallow, weakly hooked	***M. bingoensis* Yamato, 1987**
‒	Female pereopod 6, anterior lobe of coxa deep, strongly hooked	***M. nagatai* Yamato, 1987**
6	Antenna 2, flagellum strongly setose	***M. setiflagella* Yamato, 1988**
‒	Antenna 2, flagellum weakly setose	**7**
7	Antenna 1, accessory flagellum 4-articulate; male gnathopod 2, palm quadrate	***M. koreana* Stephensen, 1944**
‒	Antenna 1, accessory flagellum 2-articulate; male gnathopod 2, palm oblique	**8**
8	Male uropod 3 outer ramus weakly arched, more than 7.0 times longer than wide; anterior lobe of coxa of female pereopod 6 as deep as coxa is, strongly hooked	***M. choshigawaensis* sp. n.**
‒	Male uropod 3 outer ramus sublinear, about 5 times as long as wide; anterior lobe of coxa of female pereopod 6 depth less than coxal width, weakly hooked	***M. shimizui* (Uéno, 1940)**
9	Antenna 2 flagellum strongly setose; uropod 3 outer ramus with long setae	***M. quadridentata* Yamato, 1990**
‒	Antenna 2 flagellum weakly setose; uropod 3 outer ramus without long setae	**10**
10	Inferior antennal sinus absent	***M. pilopropoda* Hirayama, 1987**
‒	Inferior antennal sinus present	**11**
11	Maxilla 1 palp article 1 with setae; male gnathopod 2 propodus palm oblique	***M. hoshinoi* Yamato, 1990**
‒	Maxilla 1 palp article 1 without setae; male gnathopod 2 propodus palm quadrate	***M. rylovae* Bulycheva, 1955**

## Supplementary Material

XML Treatment for Melita choshigawaensis
